# Supplementary material for: Inhibition of Phenol from Entering into Condensed Freshwater by Activated Persulfate during Solar-Driven Seawater Desalination
Source: Molecules. 2022 Oct 23;27(21):7160. doi: 10.3390/molecules27217160 (PMC9657060; doi:10.3390/molecules27217160)
Supplement: Supplementary file 1 [file molecules-27-07160-s001.zip › molecules-1944879-supplementary.pdf]

Supporting Information

Inhibition of Phenol from Entering into Condensed Freshwater by Activated Persulfate during Solar-Driven Seawater Desalination

Xiaojiao Zhou [1], Ningyao Tao [1], Wen Jin [1], Xingyuan Wang [1], Tuqiao Zhang [1] and Miaomiao Ye [1,2,*]

[1] Zhejiang Key Laboratory of Drinking Water Safety and Distribution Technology, College of Civil Engineering and Architecture, Zhejiang University, Hangzhou 310058, China
[2] Donghai Laboratory, Zhoushan 316021, China
* Correspondence: yemiao008@zju.edu.cn; Tel.: +86-571-88206759

Number of Pages: 10

Number of Figures: 3

Number of Tables: 2

Number of References: 14

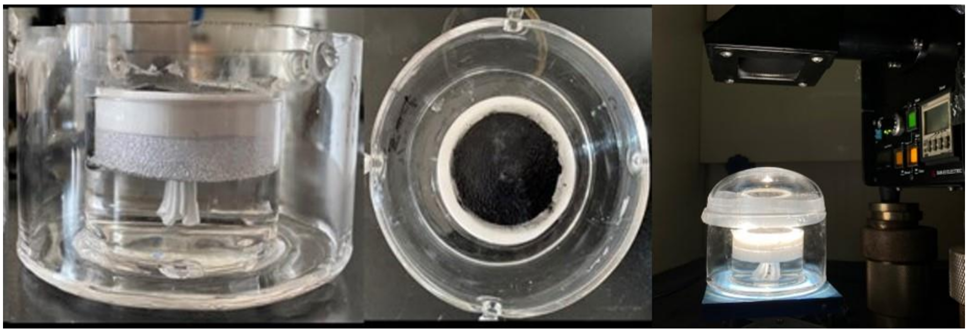

Figure S1. The actual picture of the solar evaporator.

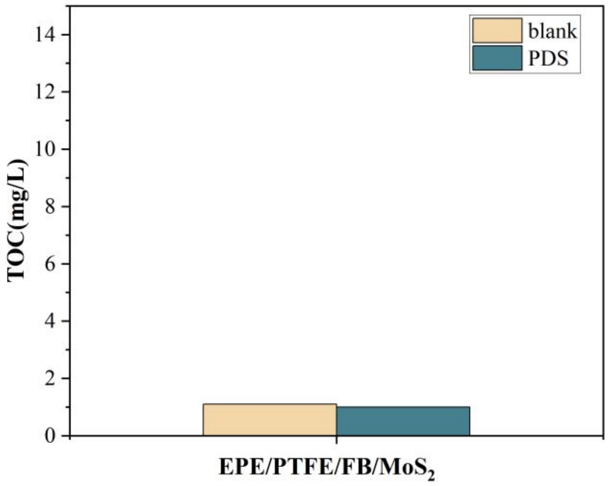

Figure S2. TOC concentration in condensed freshwater by the EPE/PTFE/FB/MoS_2 evaporator with and without addition of PDS.

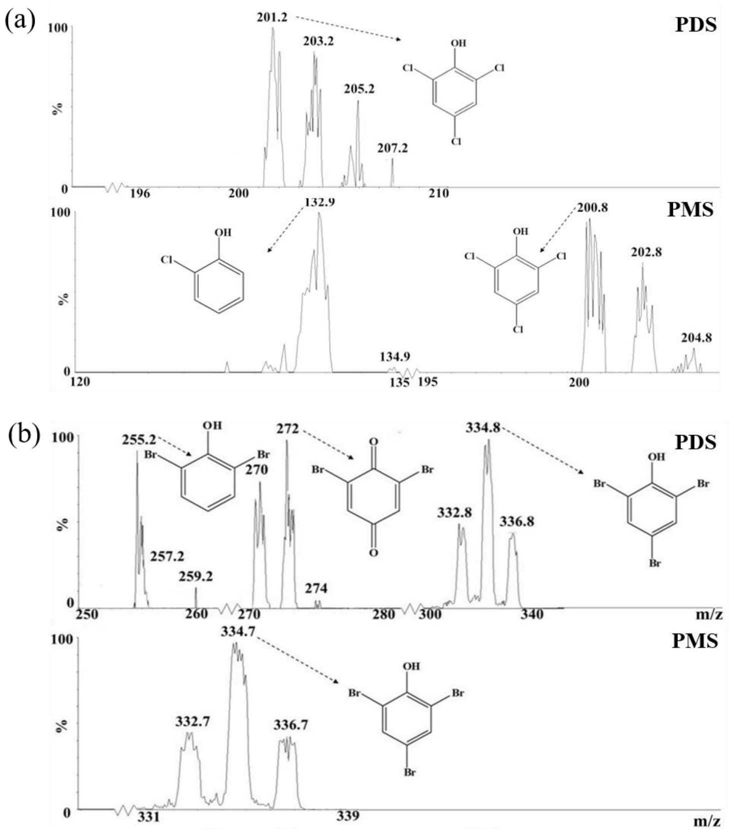
(a)
PDS
201.2
203.2
205.2
207.2
OH
Cl
PMS
132.9
134.9
200.8
202.8
204.8
%
m/z
(b)
255.2
257.2
259.2
270
272
274
Br
O
332.8
334.8
336.8
332.7
334.7
336.7

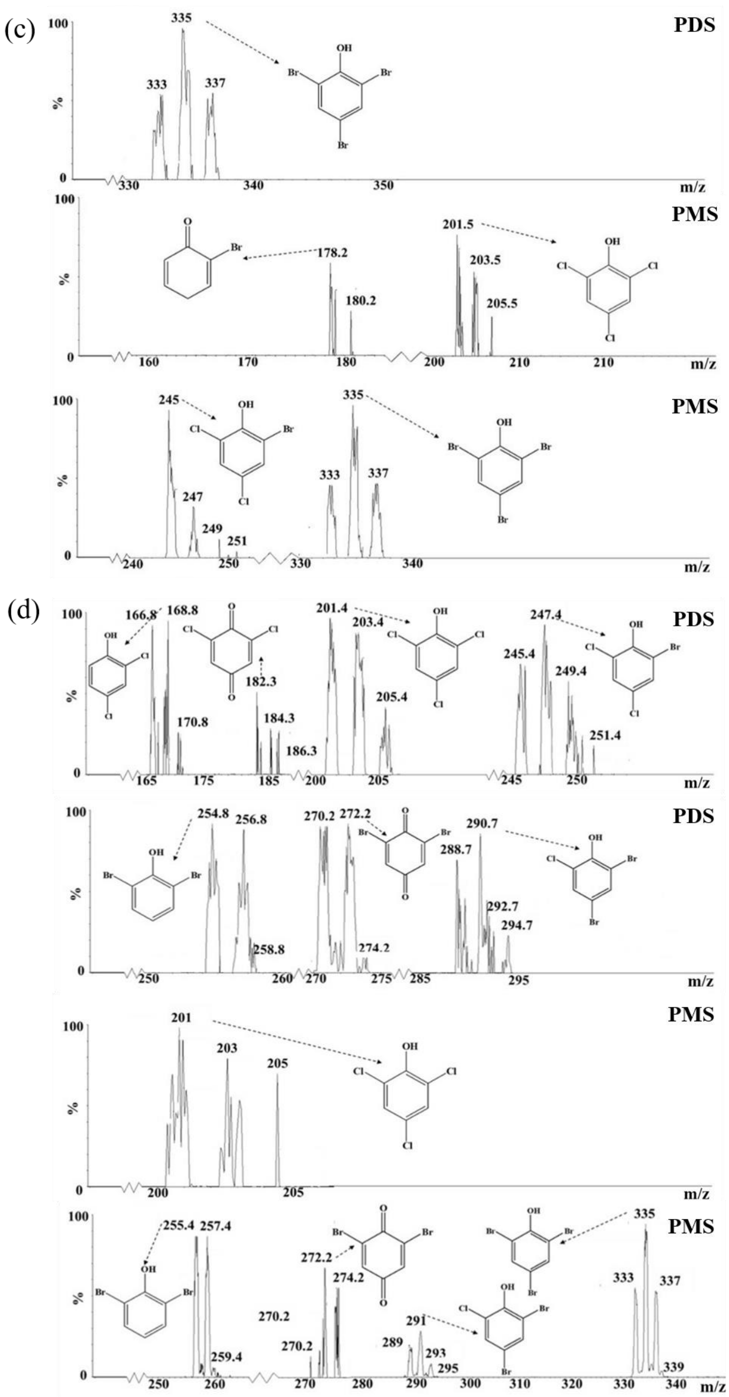

Figure S3. The halogenated distillation by-products in freshwater detected by UPLC/ESI-tqMS in (a) group 1, (b) group 2, (c) group 3, (d) group 4. (group 1: 3.5 wt% NaCl solution, group 2: 3.5 wt% NaBr solution, group 3: 3.5 wt% NaCl mixed with 0.09 wt% NaBr, and group 4: actual seawater)

Table S1. Summary of representative photothermal materials under solar irradiance

Number	Photothermal material	Solar intensity (kW/m^2)	Interface temperature (℃)	Evaporation rate (kg/m^2 h)	References
1	surface modified coconut fiber	1	69	1.37	[1]
2	PDA/PEI/PPy@PI nanofibrous membrane	1	36.4	1.43	[2]
3	laser-induced graphene/polyimide (LIG/PI) photothermal membrane	1	49	1.42	[3]
4	carbon-nanotube-embedded polyacrylonitrile nonwoven fabrics	1	42.6	1.44	[4]
5	paper fibers combined with graphite sheets	1	38	1.16	[5]
6	Ag nanoparticles modified floating carbon cloth (ANCC)	1	36.1	1.36	[6]
7	Ag_3PO_4-rGO	1	43	1.31	[7]
8	CuS/cellulose composite film	1	36.7	1.30	[8]
9	activated carbon	1	44	1.27	[9]
10	Graphene nanosheet	1	41.0	1.31	[10]
11	Graphite particles	1	44.5	1.06	[11]
12	flame-treated wood	1	43	1.05	[12]
13	polypyrrole-functionalized pomelo peel	1	54	1.22	[13]
14	black sands	1	43.4	1.43	[14]
**15**	**This work**	**1**	**42.1**	**1.35**	/

Table S2. Formation of halogenated distillation by-products in freshwater in different groups.

Group	Type	chloro/bromo by-products
1	PDS with 3.5 wt% NaCl solution	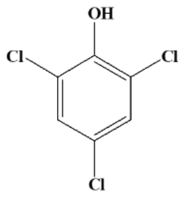
	PMS with with 3.5 wt% NaCl solution	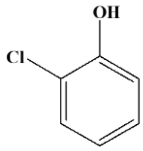 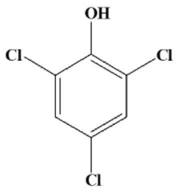
2	PDS with 3.5 wt% NaBr solution	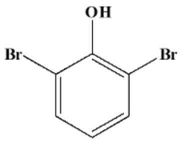 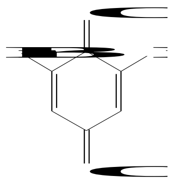 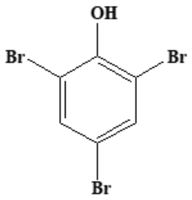
	PMS with 3.5 wt% NaBr solution	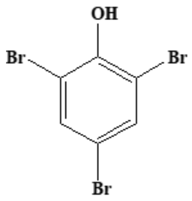
3	PDS with the mixtures of 3.5 wt% NaCl and 0.09 wt% NaBr solution	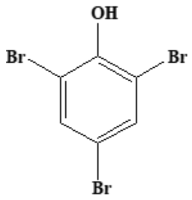
	PMS with the mixtures of 3.5 wt% NaCl and 0.09 wt% NaBr solution	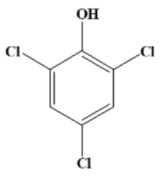 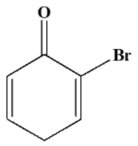 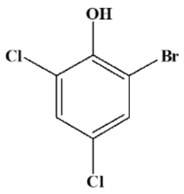 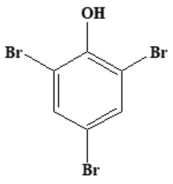

4	PDS with actual seawater	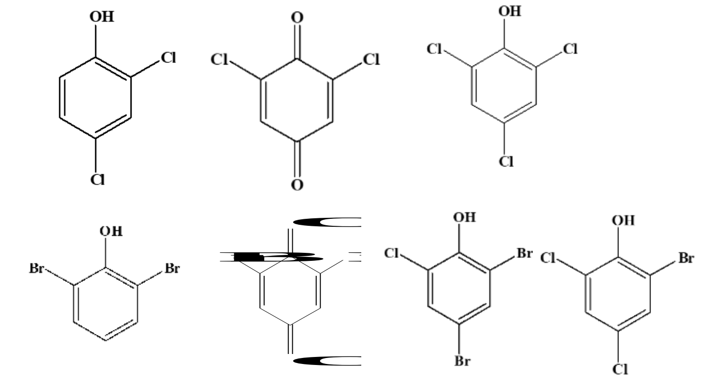
	PMS with actual seawater	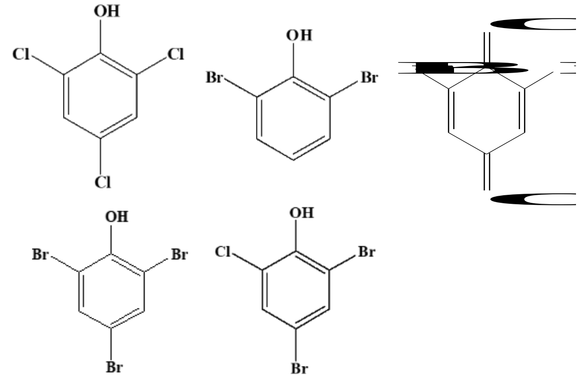

Reference

[1] Li, J.Y.; Zhou, X.; Chen, G.B.; Wang, F.; Mao, J.L.; Long, Y.; Sun, H.X.; Zhu, Z.Q.; Liang, W.D.; Li, A. Evaporation efficiency monitoring device based on biomass photothermal material for salt-resistant solar-driven interfacial evaporation. *Sol. Energy Mater. Sol. Cells*. **2021,** *222*, 9.

[2] Xu, Y.; Xu, H.B.; Zhu, Z.G.; Hou, H.Q.; Zuo, J.L.; Cui, F.Y.; Liu, D.M.; Wang, W., A mechanically durable, sustained corrosion-resistant photothermal nanofiber membrane for highly efficient solar distillation. *J. Mater. Chem. A*. **2019,** *7*, 22296-22306.

[3] Chen, Z.C.; Li, A.; Chen, X.M., Porous graphene/polyimide membrane with a three-dimensional architecture for rapid and efficient solar desalination via interfacial evaporation. *ACS Sustain. Chem. Eng.* **2020,** *8*, 13850-13858.

[4] Zhu, B.; Kou, H.; Liu, Z.X.; Wang, Z.J.; Macharia, D.K.; Zhu, M.F.; Wu, B.H.; Liu, X.G.; Chen, Z.G., Flexible and washable CNT-embedded pan nonwoven fabrics for solar-enabled evaporation and desalination of seawater. *ACS Appl. Mater. Interfaces*. **2019,** *11*, 35005-35014.

[5] Xu, Y.; Ma, J.X.; Liu, D.Q.; Xu, H.B.; Cui, F.Y.; Wang, W., Origami system for efficient solar driven distillation in emergency water supply. *Chem. Eng. J.* **2019,** *356*, 869-876.

[6] Qiao, P.Z.; Wu, J.X.; Li, H.Z.; Xu, Y.C.; Ren, L.; Lin, K.; Zhou, W., Plasmon ag-promoted solar-thermal conversion on floating carbon cloth for seawater desalination and sewage disposal. *ACS Appl. Mater. Interfaces*. **2019,** *11*, 7066-7073.

[7] Noureen, L.; Xie, Z.J.; Gao, Y.J.; Li, M.M.; Hussain, M.; Wang, K.; Zhang, L.B.; Zhu, J.T., Multifunctional Ag_3PO_4-rGO-coated textiles for clean water production by solar-driven evaporation, photocatalysis, and disinfection. *ACS Appl. Mater. Interfaces* **2020,** *12,* 6343-6350.

[8] Shang, M.Y.; Xu, S.H.; Li, J.L.; Sun, H.J.; Peng, J.; Wang, S.; Zhang, M., CuS hollow nanospheres/cellulose composite film as a recyclable interfacial photothermal evaporator for solar steam generation. *Energy Technol.* **2022,** *10,* 9.

[9] Mnoyan, A.; Choi, M.; Kim, D.H.; Ku, B.J.; Kim, H.; Lee, K.J.; Yasin, A.S.; Nam, S.; Lee, K., Cheap, facile, and upscalable activated carbon-based photothermal layers for solar steam generation. *RSC Adv.* **2020,** *10*, 42432-42440.

[10] Wu, S.H.; Gong, B.Y.; Yang, H.C.; Tian, Y.K.; Xu, C.X.; Guo, X.Z.; Xiong, G.P.; Luo, T.F.; Yan, J.H.; Cen, K.F.; Bo, Z.; Ostrikov, K.K.; Fisher, T.S., Plasma-made graphene nanostructures with molecularly dispersed F and Na sites for solar desalination of oil-contaminated seawater with complete in-water and in-air oil rejection. *ACS Appl. Mater. Interfaces*. **2020,** *12*, 38512-38521.

[11] Peng, G.L.; Ding, H.R.; Sharshir, S.W.; Li, X.J.; Liu, H.C.; Ma, D.K.; Wu, L.R.; Zang, J.F.; Liu, H.; Yu, W.; Xie, H.Q.; Yang, N., Low-cost high-efficiency solar steam generator by combining thin film evaporation and heat localization: Both experimental and theoretical study. *Appl. Therm. Eng.* **2018,** *143,* 1079-1084.

[12] Xue, G.B.; Liu, K.; Chen, Q.; Yang, P.H.; Li, J.; Ding, T.P.; Duan, J.J.; Qi, B.; Zhou, J., Robust and Low-Cost Flame-Treated Wood for High-Performance Solar Steam Generation. *ACS Appl. Mater. Interfaces* **2017,** *9*, 15052-15057.

[13] Zhang, C.; Peng, X.; Feng, N.; Yan, L.K.; Liu, Q.Q.; Zhang, D.; Gu, J.C.; Wang, W.Q.; Chen, T., Converting pomelo peel into eco-friendly and low-consumption photothermic biomass sponge toward multifunctioal solar-to-heat conversion. *ACS Sustain. Chem. Eng.* **2020,** *8*, 5328-5337.

[14] Ni, F.; Xiao, P.; Qiu, N.X.; Zhang, C.; Liang, Y.; Gu, J.C.; Xia, J.Y.; Zeng, Z.X.; Wang, L.P.; Xue, Q.J.; Chen, T., Collective behaviors mediated multifunctional black sand aggregate towards environmentally adaptive solar-to-thermal purified water harvesting. *Nano Energy* **2020,** *68*, 10.
